# Tissue- and age-dependent expression of RNA-binding proteins that influence mRNA turnover and translation

**DOI:** 10.18632/aging.100073

**Published:** 2009-07-26

**Authors:** Kiyoshi Masuda, Bernard Marasa, Jennifer L. Martindale, Marc K. Halushka, Myriam Gorospe

**Affiliations:** ^1^ Laboratory of Cellular and Molecular Biology, NIA-IRP, NIH, Baltimore, MD 21224, USA; ^2^ Department of Pathology, The Johns Hopkins University, Baltimore, Maryland 21231, USA

**Keywords:** HuR, AUF1, TIA-1, TTP, TTR-RBPs, senescence

## Abstract

Gene expression patterns vary dramatically in a tissue-specific and age-dependent manner. RNA-binding proteins that regulate mRNA turnover and/or translation (TTR-RBPs) critically affect the subsets of expressed proteins.  However, very little is known regarding the tissue- and age-dependent expression of TTR-RBPs in humans.  Here, we use human tissue arrays containing a panel of organ biopsies from donors of different ages, to study the distribution and abundance of four TTR-RBPs:  HuR, AUF1, TIA-1, and TTP.  HuR and AUF1 were expressed with remarkably similar patterns.  Both TTR-RBPs were present in high percentages of cells and displayed elevated intensities in many age groups and tissues, most notably in the gastrointestinal and reproductive systems;  they were moderately expressed in the urinary and immune systems, and were almost undetectable in muscle and brain.  TIA-1 was also abundant in many tissues and age groups;  TIA-1 was expressed at high levels in the gastrointestinal, immune, urinary, and reproductive systems, and at low levels in brain and muscle.  By contrast, TTP-expressing cells, as well as TTP signal intensities declined with advancing age, particularly in the immune, nervous, and muscular systems;  however, TTP levels remained elevated in the gastrointestinal tract.  The widespread abundance of HuR, AUF1, and TIA-1 throughout the body and in all age groups was in stark contrast with their declining levels in human diploid fibroblasts (HDFs) undergoing replicative senescence, a cultured-cell model of aging. Conversely, TTP levels increased in senescent HDFs, while TTP levels decreased with advancing age.  Our studies provide a framework for the study of human TTR-RBP function in different tissues, throughout the human life span.

## Introduction

Throughout the lifetime of an organism, gene expression programs change dynamically.  The specific subsets of proteins expressed at each point in time allow cells to carry out long-term functions, such as those needed during development and differentiation, and short-term adaptive changes, including responses to acute environmental or hormonal stimuli.  The gene ex-pression patterns that characterize each tissue at different developmental stages are strongly regulated at the transcriptional level.  Transcription factors (TFs) such as FOXO (forkhead box), PPAR (peroxisome proliferator-activated receptor)γ, p53, C/EBP (CCAAT/enhanncer-binding protein), as well as by chromatin remodeling factors such as MRG and HDACs have been implicated in aging and age-related processes [1-6].

However, gene expression patterns are also potently regulated by RNA-binding proteins (RBPs), which control post-transcriptional processes such as pre-mRNA splicing, and mRNA cytoplasmic export, turnover, storage, and translation [7-10].  Unlike TFs, much less is known about the role of RBPs in aging and age-related events.  A subset of RBPs which function as *t *ranslation and *t *urnover *r *egulatory (TTR) RBPs is of particular interest, since numerous genes implicated in age-related processes encode mRNAs that are labile and/or subject to translational control [11].  Examples of age-related proteins whose mRNAs are targets of TTR-RBPs include p16^INK4^, p21^CIP1^, cyclins (D1, E, A, B1, and H), cdk1 (cyclin-dependent kinase 1), CAK (cdk-activating kinase), amyloid precursor protein (APP), endothelin-1, fibronectin, interleukin (IL)-1, Cu,Zn- and Mn-superoxide dismutase (SOD), growth arrest- and DNA damage-inducible (GADD)45, plasminogen activator inhibitor (PAI)-1 and PAI-2, collagenase, granulocyte macrophage-colony-stimulating factor (GM-CSF) and M-CSF, p53, bcl-2, p33^ING1^, c-fos, catalase, E2F-1,-2, DP-1, elastin, thymidine kinase, insulin growth factor (IGF)-II, dihydrofolate reductase, PCNA, ribonucleotide reductase, and histones (reviewed in [11]). Here, we use arrays of human tissue biopsies to study the tissue distribution of four major TTR-RBPs as a function of age:  HuR (human antigen R), AUF1 (AU-binding factor 1, also called heterogenous ribonucleoprotein D or hnRNP D), TIA (T-cell intracellular antigen)-1, and TTP (tristetraprolin).

HuR is the ubiquitously expressed member of the embryonic lethal abnormal vision (ELAV)/Hu protein family, which also comprises the primarily neuronal proteins HuB, HuC, and HuD [12].  Through its RNA-recognition motifs (RRMs), HuR binds to numerous mRNAs bearing AU- and U-rich sequences and stabilizes and/or modulates their translation [12-14].  Many HuR target mRNAs encode proteins important for cell growth, proliferation, and survival, as well as for the immune and stress responses [11,12,15-17]. Examples include mRNAs that encode cyclins (A, B1, E, D1), c-fos, c-myc, vascular endothelial growth factor (VEGF), hypoxia-inducible factor-1α (HIF-1α), prothymosin-α, cyclooxygenase (COX)-2, tumor necrosis factor (TNF)-α, and several interleukins (reviewed in [11,12]).

AUF1 comprises four proteins that arise from alternative splicing (p37, p40, p42, p45) and shuttle between the nucleus and the cytoplasm [18,19].  AUF1 has also been implicated in several distinct post-transcriptional processes.  Originally found to promote mRNA decay, as revealed in studies using a variety of cell systems [20-23], in some instances AUF1 has been shown to enhance mRNA stability and to promote translation [21,24-26].  All of the AUF1 isoforms contain two RRMs through which they bind to a select group of mRNAs, including many mRNAs that encode stress-response, immune, and proliferative proteins such as p21, cyclin D1, myc, fos, GM-CSF, TNF-α, IL-3, parathyroid hormone (PTH), and GADD45α [21-24,27,28].

TIA-1 and the TIA-1-related protein (TIAR) are believed to play general roles as translational repressors in response to environmental stress agents (heat, oxidants, hyperosmolarity, etc.) [29-33]. Such damaging factors trigger the aggregation of TIA-1 into discrete cytoplasmic foci called stress granules (SGs), wherein mRNAs are thought to be stored transiently and subsequently sorted into the translation and degradation machineries.  Many TIA-1/TIAR target mRNAs, often C-rich or U-rich [33,34], are translationally repressed when they are associated with TIA-1/TIAR and become translated upon dissociation from TIA-1 [27,33,35].  TIA-1 regulates the translation of mRNAs encoding TNF-α, COX-2, and several other transcripts bearing a TIA-1 motif [29,31,36].

The product of the ZFP-36 gene, TTP is also known as TIS11, Nup475, and GOS24.  TTP binds mRNAs through two tandem CCCH zinc finger motifs and promotes their decay [37].  TTP target mRNAs typically contain the AU-rich sequence UUAUUUAUU, although TTP also binds to tandem repeats of the shorter sequence UUAU [38,39].  TTP is induced as an immediate-early response gene in response to inflammatory mediators and growth factors in many cell types, including T cells, macrophages, and fibroblasts [40-43].  By destabilizing one of its target transcripts, TNF-α mRNA, TTP reduced inflammation [40].  TTP also induced the decay of other mRNAs, such as those encoding GM-CSF, COX-2, IL-3, IL-10, and interferon-γ [44-48].

We previously reported that cultured human diploid fibroblasts (HDFs) undergoing replicative senescence showed reduced levels of HuR, which contributed to the diminished expression of cyclin A, cyclin B1, and c-fos in senescent HDFs [49], and also showed reduced AUF1 levels, which contributed to elevating p16 abundance, a senescence marker in this cell system [50].  Fibroblasts explanted from donors of different ages, then briefly expanded in culture, also showed a moderate reduction in HuR levels as the age of the donor increased [49].  There is agreement that senescent HDFs recapitulate some features of cells from the elderly and there is broad support for the notion that cellular senescence constitutes a tumor suppressive mechanism, particularly in young and middle-aged individuals.  However, the links between cellular senescence and aging or age-related processes are considerably weaker.

A systematic study of TTR-RBPs in human tissues has not been performed to-date.  Yet many age-related genes are encoded by mRNAs which are labile and/or subject to translational regulation.  Therefore it was important to investigate the expression of TTR-RBPs, particularly HuR, AUF1, TIA-1, and TTP, in a panel of human tissues spanning different ages.  We quantified both the percentages of TTR-RBP-positive cells and their relative intensity as a function of individual donor age.  This analysis provided a wealth of information;  salient among it was the finding that HuR, AUF1, and TIA-1 remained highly expressed in many aging tissues, particularly in gastrointestinal (GI) and reproductive organs, despite their reduced abundance in replicative senescent HDFs.  It was also interesting to discover that TTP expression pattern was opposite to that of other TTR-RBPs, increasing with replicative senescence and decreasing in many tissues with advancing age. These findings reveal an important discordance between TTR-RBP levels during replicative senescence and those present during *in vivo* aging, and provide a valuable framework of tissue- and age-dependent TTR-RBP expression for future *in vivo* analyses.  Furthermore, they suggest that HuR, AUF1, and TIA-1 likely play important roles in maintaining tissue homeostasis with advancing age.

## Results

### HuR, AUF1, and TIA-1 levels decrease and TTP levels increase during replicative senescence

**Figure 1.**
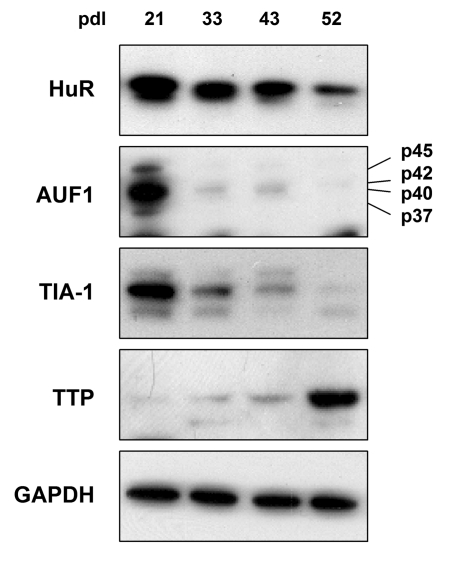
TTR-RBP expression in WI-38 human diploid fibroblasts (HDFs). WI-38 HDFs were cultured for extended population doublings (pdls), until they reached senescence at ~pdl 52.  The abundance of TTR-RBPs HuR, AUF1 (all four isoforms indicated), TIA-1, and TTP was assessed by Western blot analysis. GAPDH signals were included as a loading control.

To investigate the relative changes in TTR-RBP levels occurring with replicative senescence and with increasing age, we began by assessing TTR-RBP abundance in HDFs.  Early-passage, proliferating (‘young') WI-38 HDFs were cultured until they ceased cell division and became senescent, as previously reported [51].  At the indicated population doublings (pdls), protein lysates were prepared and the levels of HuR, AUF1, TIA-1, and TTP were detected by Western blot analysis.  As shown in Figure 1, HuR, AUF1, and TIA-1 were most abundant in early-passage HDFs (pdl 21), declining thereafter, as previously reported for HuR and AUF1 [49,50].  The levels of AUF1 and TIA-1 declined markedly by pdls 33 and 43, becoming virtually undetectable by pdl 52, when cells were senescent, while the decline in HuR levels was slower and less pronounced.  Unexpectedly, the expression pattern of TTP was just the opposite, displaying extremely low levels in early-passage cells and increasing dramatically as WI-38 HDFs reached senescence.  The levels of GAPDH were measured to ensure equal loading.

### HuR levels remain elevated in numerous tissues with advancing age

In order to study the tissue- and age-dependent expression patterns of TTR-RBPs, we obtained tissue arrays which contained a panel of healthy tissue biopsies from human donors of different ages (fetal through adult; Array II, BioChain Institute;  FDA 35, Pantomics, Inc.).  The tissue arrays were probed with an anti-HuR antibody in order to visualize HuR signals in the different tissues; the slides were then scanned and the digital images were analyzed as explained in the Methods section.  For the analysis, the donors' ages were grouped as follows:  fetal (*F*), young (*Y*, birth to 30 years of age), middle-aged (*M*, 30 to 60 years of age), or old (*O*, over 60 years of age).  The exact ages and tissue types of the biopsies analyzed in both arrays are listed (Supplementary Table 1).  The signals of each spot on the array were measured in two ways:  by counting the percentage area of positive cells (*% HuR positive*) and by measuring the intensity of the signals in the positive cells (*Intensity*).  These values were calculated from the digitized images using a color deconvolution algorithm to identify diaminobenzidine (DAB, "brown") positivity in defined regions of interest (ROI) [52].  The data were tabulated showing the number of samples analyzed in each age group in  parenthesis, and the average percentage values with the color scheme shown;  in some cases, a tissue in a given age group was not available in either of the arrays studied (n.a.). Negative immunohistochemis-try signals are shown in the Supplementary Figure 1.

**Table 1.**
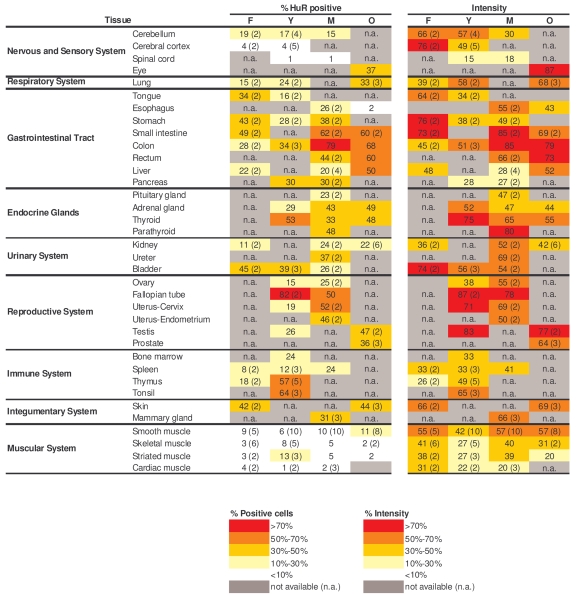
Quantitation of HuR signals in human tissue microarrays. Shown are the percentages of positive area (‘% HuR positive') and the signal strength (‘Intensity') in samples from a range of tissue types and age groups.  When multiple biopsies were quantified in a given tissue and age group, the average value is shown and the number of tissues examined is indicated in parenthesis.  Values were calculated as explained in the Methods section.

As observed, HuR-positive cells were detected in virtually all tissues and age groups (Table 1, left columns), but were very low in neuronal and muscle tissues.  The numbers of HuR-positive cells remained relatively unchanged with increasing age in most tissues examined, increasing with age only in the lung and in the gastrointestinal (GI) tract (small intestine, colon, rectum).  When HuR intensities were compared (Table 1, right columns), there was little loss in HuR abundance with advancing age in most tissues examined, declining only in the nervous system.  Most tissues showed little change in HuR levels across age groups (e.g., skeletal muscle, skin, and reproductive and urinary systems), although an increase was observed again in the lung.  It is worth noting that strong HuR sig- nals were seen throughout age groups in the GI, reproductive, and urinary systems.

**Figure 2.**
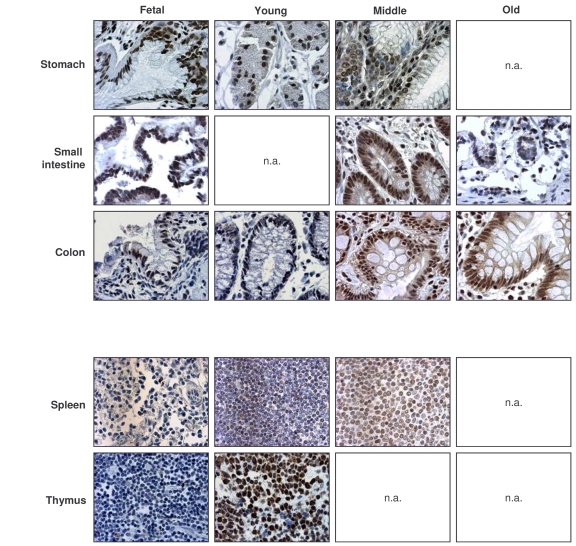
Immunohistochemical detection of HuR across tissue types and age groups. Representative HuR signals in photomicrographs taken from the indicated tissue sections from human tissue arrays.  Images are shown at ×200 magnification.

Representative photographs from the tissue array are shown (Figure 2).  Samples from the GI tract (stomach, small intestine, colon) and the immune system (spleen, thymus) were selected, as the levels and age-dependent changes in these tissues were particularly interesting for all TTR-RBPs examined.  In summary, HuR was ubiquitously expressed in a broad range of human tissues and showed strong intensity despite advancing age.  These results contrasted with the loss of HuR expression seen in senescent HDFs ([49], Figure 1), and suggest that HuR remains functionally important with advancing age.

**Table 2.**
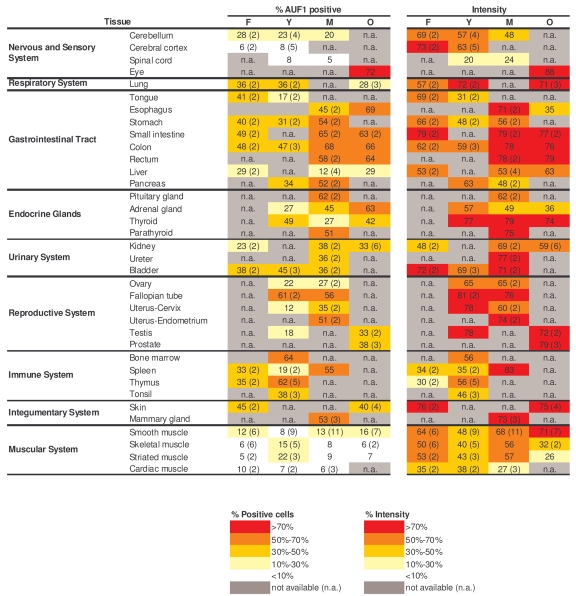
Quantitation of AUF1 signals in human tissue microarrays. Shown are the percentages of positive area (‘% AUF1 positive') and the signal strength (‘Intensity') in samples from a range of tissue types and age groups.  When multiple biopsies were quantified in a given tissue and age group, the average value is shown and the number of tissues examined is indicated in parenthesis.  Values were calculated as explained in the Methods section.

### AUF1 expression is ubiquitous and overall abundant, increasing with age in the immune system**

The analysis of AUF1 in tissue arrays was performed as described above for HuR.  Interestingly, the relative percentages of AUF1-expressing cells throughout the age groups, as well as the relative intensities of AUF1 signals were rather similar to those seen for HuR (compare Table 2 with Table 1);  the tight correlation between AUF1 and HuR signals was quantified (Supplementary Figure 2).  A similar correlation between AUF1 and HuR expression levels was noted by Lu and Schneider, who compared their relative abundance in adult mouse tissues [53].

**Figure 3.**
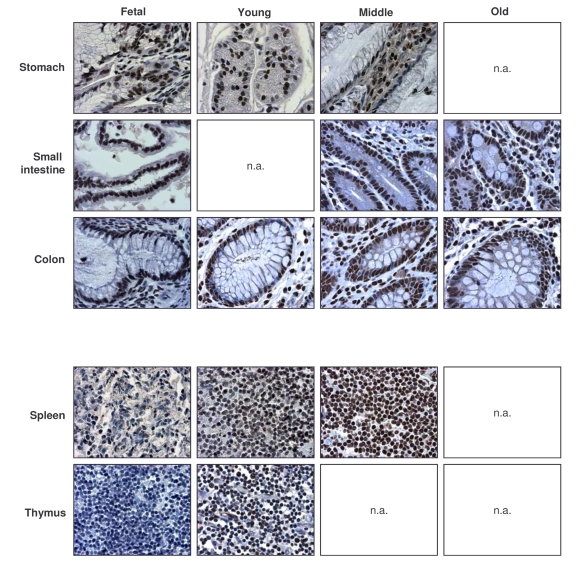
Immunohistochemical detection of AUF1 across tissue types and age groups. Representative AUF1 signals in photomicrographs taken from the indicated tissue sections from human tissue arrays.  Images are shown at ×200 magnification.

AUF1-positive cells were detected in all tissues examined, but were low in muscle, and high in the GI and immune systems.  AUF1 abundance increased with age in the immune system and was overall high in the lung, GI tract, and urinary and reproductive systems. Representative photographs of AUF1 expression in the GI and immune tissues are shown in Figure 3.  As seen with HuR, there was discordance between the steep decline in AUF1 levels in senescent HDFs and the markedly elevated AUF1 levels seen in tissues from elderly donors (Table 2).  These findings support the notion that AUF1 also plays a functional role in the tissues of elderly individuals.

**Table 3.**
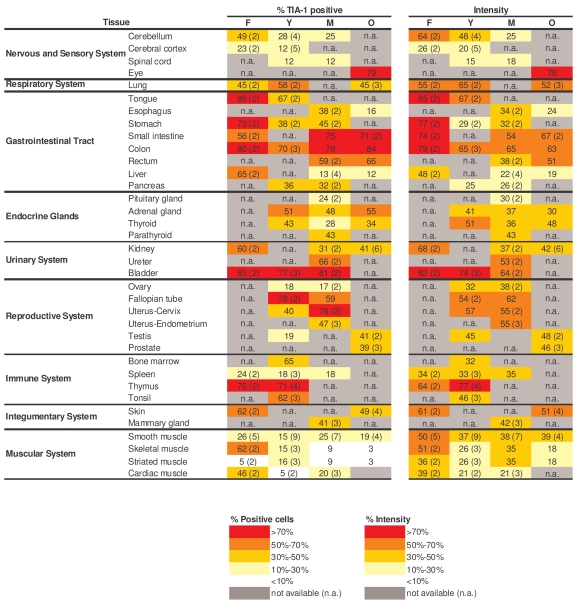
Quantitation of TIA-1 signals in human tissue microarrays. Shown are the percentages of positive area (‘% TIA-1 positive') and the signal strength (‘Intensity') in samples from a range of tissue types and age groups.  When multiple biopsies were quantified in a given tissue and age group, the average value is shown and the number of tissues examined is indicated in parenthesis.  Values were calculated as explained in the Methods section.

### Broad expression of TIA-1 across tissues and age groups

**Figure 4.**
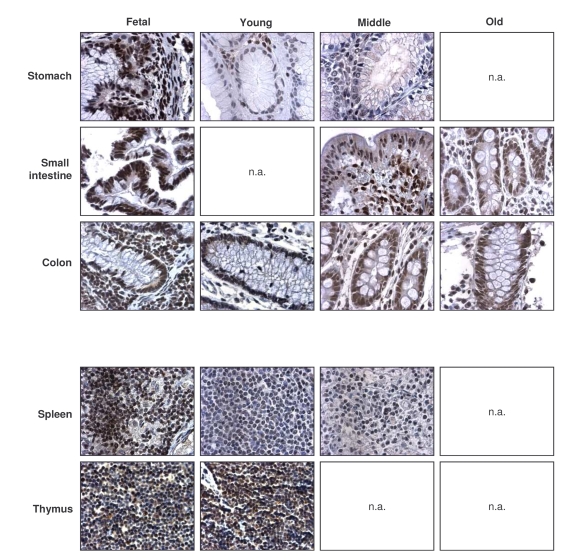
Immunohistochemical detection of TIA-1 across tissue types and age groups. Representative TIA-1 signals in photomicrographs taken from the indicated tissue sections from human tissue arrays.  Images are shown at ×200 magnification.

While TIA-1 also displayed a ubiquitous distribution, TIA-1-positive cells showed a moderate decline in some tissues of the GI and muscle systems (Table 3, left columns).  Unlike HuR and AUF1, the relative intensity of TIA-1 in several tissues declined with advancing age, as seen in the endocrine, urinary, and muscle systems.  Despite a moderate decline in TIA-1 signals in the GI tract, its levels remained relatively high here and were also elevated in all age groups in the respiratory, immune, and reproductive systems (Table 3, right columns).  Sample photographs of TIA-1 signals in immune and GI specimens are shown in Figure 4. Once again, TIA-1 followed a time-dependent pattern of expression in tissues that was largely opposite to what was seen in cultured WI-38 HDFs advancing towards senescence: highly expressed *in vivo* (Table 3, Figure 4), progressive-ly lower until almost undetectable *in vitro* (Figure 1). These observations suggest that TIA-1 may also be important for regulating gene expressionwith advancing age.

### General decline in TTP-expressing cells and TTP levels with advancing age

**Figure 5.**
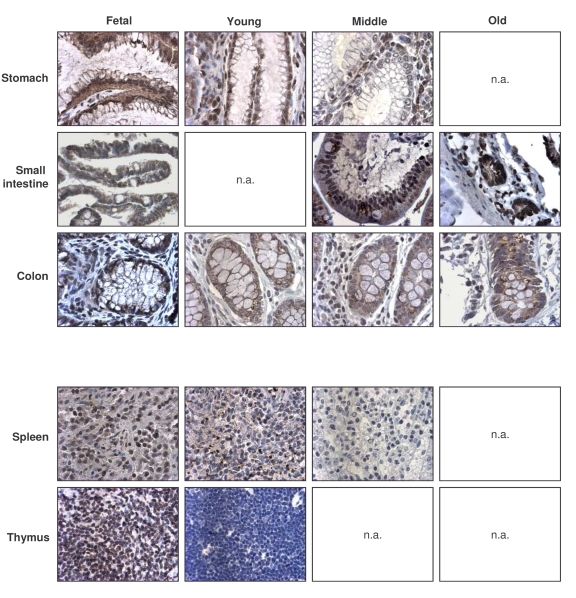
Immunohistochemical detection of TTP across tissue types and age groups. Representative TTP signals in photomicrographs taken from the indicated tissue sections from human tissue arrays.  Images are shown at ×200 magnification.

Like TIA-1, the numbers of TTP-expressing cells were highest in the fetal (F) group, generally decreasing in older groups (Table 4, left columns).  Exceptions to this pattern were the GI and endocrine systems, where TTP-positive cell numbers remained constant across age groups, and the reproductive tissues, where TTP-positive cells increased with advancing age. TTP intensities also generally declined across tissue types when examining progressively older donors (Table 4, right columns).  Representative micrographs from the GI and immune systems are shown (Figure 5).  The dis-agreement between replicative senescence and *in vivo* aging was also seen with TTP, as senescent cells expressed increasingly higher TTP, while advancing age progressively lowered the number of TTP-expressing cells and TTP abundance per cell.  Although TTP levels can be induced by a variety of stimuli, the constitutive TTP expression decreased markedly with advancing age.

**Table 4.**
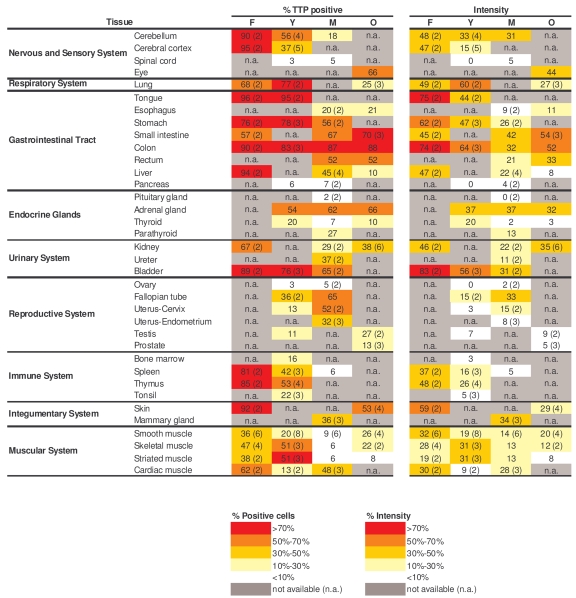
Quantitation of TTP signals in human tissue microarrays. Shown are the percentages of positive area (‘% TTP positive') and the signal strength (‘Intensity') in samples from a range of tissue types and age groups.  When multiple biopsies were quantified in a given tissue and age group, the average value is shown and the number of tissues examined is indicated in parenthesis.  Values were calculated as explained in the Methods section.

## Discussion

Our results reveal an interesting discordance between the levels of four TTR-RBPs in human fibroblasts undergoing replicative senescence and their levels in tissues from individuals of increasing age.  In WI-38 cells, senescence potently lowered HuR, AUF1, and TIA-1 levels, while it increased TTP abundance (Figure 1).  Accordingly, we hypothesized that the levels of HuR, AUF1, and TIA-1 might also decline with aging, while TTP levels might increase.  Using a robust method to quantify immunohistochemical signals present in different tissue types and age groups, we discovered that *in vivo*, these TTR-RBPs were expressed in precisely the opposite pattern: HuR, AUF1, and TIA-1 remained highly abundant with advancing age, in some cases even increasing their expression, while TTP levels generally decreased in the aged groups (compare Figure 1 with Tables 1-4).  This discovery was somewhat surprising,  given the wide use of HDFs as an *in vitro* model of aging and the broad agreement that senescent cells arise in normal tissues with aging *in vivo*, as discussed elsewhere [54].  However, since senescent cells are terminally arrested and may be cleared by immune cells, perhaps they are underrepresented in the tissues examined here.  Additionally, key differences exist between cultured HDF senescence and *in vivo* cellular senescence. For example, cultured HDFs are exposed to chronic levels of damaging stimuli such as supraphysiologic oxygen and overabundant growth factors, possibly triggering a persistent stress response that could elevate TTP levels and lower HuR, AUF1, and TIA-1 levels.  Conversely, a more physiologic setting would cause stress conditions of different type and magnitude in live organs, possibly impacting on TTR-RBP abundance.  While further experiments are needed to discern among these possibilities, our findings lead us to join many other laboratories in questioning the extent to which senescent HDFs recapitulate features of *in vivo* aging.

A systematic analysis of TTR-RBP expression in human tissues has not yet been performed.  To carry out such an analysis, we obtained tissue arrays that contained a wide range of human tissue biopsies from different aged subjects (Methods);  in them, we studied TTR-RBP levels using an immunohistochemical analysis method of color deconvolution that was recently adapted to tissue array analysis [52,55].  Our examination of HuR, AUF1, TIA-1, and TTP expression by immunohistochemistry showed that these proteins were expressed ubiquitously and in high abundance among many tissues across age groups (Tables 1-4).  Lu and Schneider [53] examined systematically the expression of several TTR-RBPs in adult mice.  They reported that HuR was expressed in numerous tissues, including intestine, thymus, spleen, and liver, while it was almost undetectable in brain, heart, lung, kidney, and skeletal muscle [53].  This tissue distribution is in agreement with our findings (Table 1, Figure 2), although in some human tissues, such as liver and lung, a moderate percentage of cells also expressed HuR, in some cases with high intensities.  The same authors showed that mouse AUF1 was expressed in highest abundance in thymus and spleen, but was also detectable in brain, testis, ovary, and uterus, intestine, and lung.  Although the levels of AUF1 in adult brain (M, O) could not be examined, the tissue distribution of AUF1 in mouse agrees largely with that seen in human.  Lu and Schneider used Western blot analysis to visualize AUF1, which allowed them to examine tissue-specific differences in isoform abundance [53].  This assessment was not possible on tissue arrays, since isoform-specific antibodies for immunohistochemistry are not yet available.  However, our analysis yielded other valuable information, such as the predominantly nuclear localization of AUF1 and its localization in specific cell types within a given organ (Figures 2-5 and data not shown).

By employing western blot analysis, Beck and coworkers [56] showed that TIA-1 mRNA and protein were expressed in mouse brain, spleen, and testis, but not in heart, lung, liver, skeletal muscle, or kidney.  Our results indicate that human TIA-1 was expressed in a more ample range of tissues, as we also detected high percentages of TIA-1-positive cells in the GI, urinary, and endocrine systems, and we found generally elevated TIA-1 signals among the different age groups (Table 3).  The levels of TTP have also been examined in adult mouse, with high levels of TTP protein expressed in the liver, testis, and ovaries, as well as in macrophages [53,57].  In human tissues, we also detect TTP in these organs, but again find a broader tissue distribution for TTP, with high percentages of cells expressing TTP and high TTP signals in the urinary and muscular systems, and especially in the GI tract (Table 4, Figure 5).

HuR has been implicated in numerous cell functions. Among the four TTR-RBPs studied, HuR is most tightly linked with proliferation.  Binding of HuR to mRNAs encoding cyclin A, cyclin B1, and c-fos led to their stabilization and/or increased translation, in turn accelerating cell division [49,58,59].  In keeping with this function, HuR was low in senescent HDFs (Figure 1) and contributed to their terminally arrested phenotype [49]. Given this evidence, the finding that HuR was highly expressed in many adult tissues (M, O) was unexpected.  HuR could contribute to the division of epithelial cells from the GI tract, but it likely does not exert this function in many other tissues, such as the lung, reproductive organs, and urinary system, which are populated by many non-dividing cells.  Besides proliferation, HuR was shown to have a broad pro-survival function, by binding to mRNAs encoding anti-apoptotic proteins like prothymosin α, sirtuin 1 (SIRT1), and bcl-2, and enhancing their expression [17,60,61].  Additionally, HuR's promotion of angiogenesis has been linked to its positive influence on the expression of HIF-1α and VEGF [62,63].  Perhaps the elevated abundance of HuR in post-mitotic cells helps to carry out an anti-apoptotic function and to ensure sufficient oxygen supply in terminally differentiated tissues.

All four TTR-RBPs have been linked to the immune response.  HuR function increased following mouse and human activation of macrophages and T cells [64-67].  In turn, HuR stabilized and/or modulated the translation of target mRNAs encoding numerous cytokines, such as TNF-α, IL-6, IL-13, interferon γ, and GM-CSF.  AUF1 also targets many of the same cytokine mRNAs, but it additionally downregulates IL-1β and IL-10 in immune cells [68-71].  Moreover, as AUF1-knockout mice were unable to degrade mRNAs encoding proinflammatory cytokines such as TNF-α and IL-1β, LPS treatment led to severe endotoxic shock [68].  TIA-1 also limits inflammation, at least in part by binding to the TNF-α mRNA and inhibiting TNF-α translation.  Thus, TNF-α was more highly expressed in macrophages isolated from TIA-1 knock-out mice than in those isolated from wild type mice [72]. Likewise, TTP limits inflammation by reducing the stability of GM-CSF, IL-2, and IL-3 mRNAs [44, 73, 74].  Therefore, TTP-/- mice develop severe autoimmune dysfunction, myeloid hyperplasia, and inflammatory arthritis, due to deregulated TNF-α and GM-CSF levels [57]. In human immune organs, we observed a strong constitutive presence of HuR, AUF1, and TIA-1 across the age groups studied,  while  TTP abundance declined with increasing age.  While samples from the oldest donor group were unavailable on this panel of tissues, our findings suggest that multiple TTR-RBPs likely contribute to maintaining the delicate balance that exists between promoting and inhibiting cytokine production. Taken together, we propose that these TTR-RBPs help to maintain immune homeostasis throughout human life.

In closing, cancer is among the most prominent age-related diseases, and there is increasing recognition that TTR-RBPs can modulate oncogenesis [75, 76].  The pro-malignant influence of HuR and AUF1 is well established, and numerous cancer-related mRNA targets for these TTR-RBPs have been identified [15,28].  While TIA-1 can suppress the expression of cancer-related genes such as COX-2 [36], TIA-1's involvement in cancer is less well understood.  Interestingly, suppression of TTP expression in many cancer types correlated closely with the tumorigenic phenotype and with patient prognosis [77], suggesting that TTP could have tumor suppressor function.  In light of our findings that HuR and AUF1 are elevated while TTP levels decline in tissues from aged donors, we postulate that the higher HuR and AUF1 and lower TTP could contribute to the increased incidence of cancer seen with advancing age.

While the links between senescence and aging remain to be clarified, this analysis reveals interesting distribution patterns for TTR-RBPs across tissues and age groups.  Questions for future consideration include the influence of tissue type and donor age on the subcellular localization of TTR-RBPs and their post-translational modification, as these two parameters profoundly influence the metabolism of target mRNAs.  As we work towards addressing these queries, our findings provide a framework to study the possible involvement of TTR-RBPs in age-related processes, including the loss of physiologic function and the onset of diseases associated with advancing age.

## Methods

Cell culture and treatment ***.***  WI-38 human diploid fibroblasts (Coriell Cell Repositories) were maintained in Dulbecco's modified Eagle medium (DMEM) (Invitrogen) supplemented with 10% (vol/vol) bovine calf serum (HyClone), 50 μg/ml streptomycin and penicillin, 0.1 mM nonessential amino acids, and 40 μM glutamine in a 5% CO_2_ incubator.

Western blot analysis .  Whole-cell extracts were prepared as described previously [61].  Proteins were resolved by 12% sodium dodecyl sulfate (SDS)-poly-acrylamide gel electrophoresis and transferred onto polyvinylidene difluoride membranes.  Monoclonal antibodies recognizing HuR (3A2; sc-5261) and GAPDH (6C5; sc-32233) as well as polyclonal antibodies recognizing TIA-1 (C-20; sc-1751) were from Santa Cruz Biotechnology; polyclonal antibodies recognizing AUF1 (ab61193) or TTP (ab33058) were from Abcam. After secondary-antibody incubations, signals were detected by enhanced chemiluminescence (Amersham).

Immunohistochemistry . Immunohistochemistry was performed with human adult and fetal normal tissue (Array II, BioChain Institute, Inc., Hayward, CA, and Pantomics, Inc., San Francisco, CA). The array slides were subjected to heat-induced epitope retrieval, incubation with primary antibody, and detection with the LSAB+ system (Dako, Carpinteria, CA, USA).  A monoclonal anti-HuR antibody (Molecular Probes Inc., Eugene, OR,   USA) was used at 0.2 μg/ml.  A polyclonal anti-AUF1 antibody (Abcam) was used at 1:2000 dilution, a polyclonal anti-TIA1 antibody (Santa Cruz) was used at 1:200 dilution, and a polyclonal anti-TTP antibody (Abcam) was used at 1:1000 dilution.

Slide scanning and image analysis of tissue arrays . Stained tissue sections were imaged at ×200 total magnification using a ScanScope CS system (Aperio, Vista, CA).  Whole-slide images were segmented into individual, 24-bit color core image files (TIFF) using TMALab software (Aperio) for further analysis.  Using ImageJ-based macros, regions of interest (ROI) were selected for each tissue microarray spot to exclude folded tissues and inappropriate tissue regions [52].  For example, for gastrointestinal tissues, only the epithelial cell layer was selected as the ROI, while muscular layers were excluded.  Color deconvolution was then used to separate the dye contribution at each pixel in a given image's ROI; a count of all pixels above an arbitrary threshold was determined in order to exlude background staining and to establish a mean threshold of staining.  These values were used to generate the intensity value and to calculate the "% positivity" by dividing the total ROI pixel count by the DAB positive pixel count in the ROI. The values were further classified according to age:  fetal (*F*), young (*Y*, birth to 30 yr-old), middle-aged (*M*, 30- to 60 yr-old), or old (*O*, over 60 y), and averaged the scores within each group.

## Supplementary data

Supplementary Figure 1 Background immunohistochemical signals in tissue microarrays, without incubating with primary antibody. All other steps were the same as those used to visualize HuR, AUF1, TIA-1, TTP (Figures 2-5) and prepare Tables 1-4.

Supplementary Figure 2 Correlation between the percentage positive signals for AUF1 compared with HuR, TIA-1, TTP. Taking the middle-aged samples, the correlations between positive signals within a tissue were compared. Correlation coeficients (R2) indicate that the strongest correlation was seen between HuR and AUF1. In other age groups, AUF1 and HuR also correlated most strongly (not shown).

Supplementary Table 1 Collection of tissue biopsies available in both tissue microarrays combined. (M), male; (F), female. y, years old.
