# Supplementary figures and images for: Tissue- and age-dependent expression of RNA-binding proteins that influence mRNA turnover and translation
Source: Aging (Albany NY). 2009 Jul 26;1(8):681–98. doi: 10.18632/aging.100073 (PMC2806049; doi:10.18632/aging.100073)

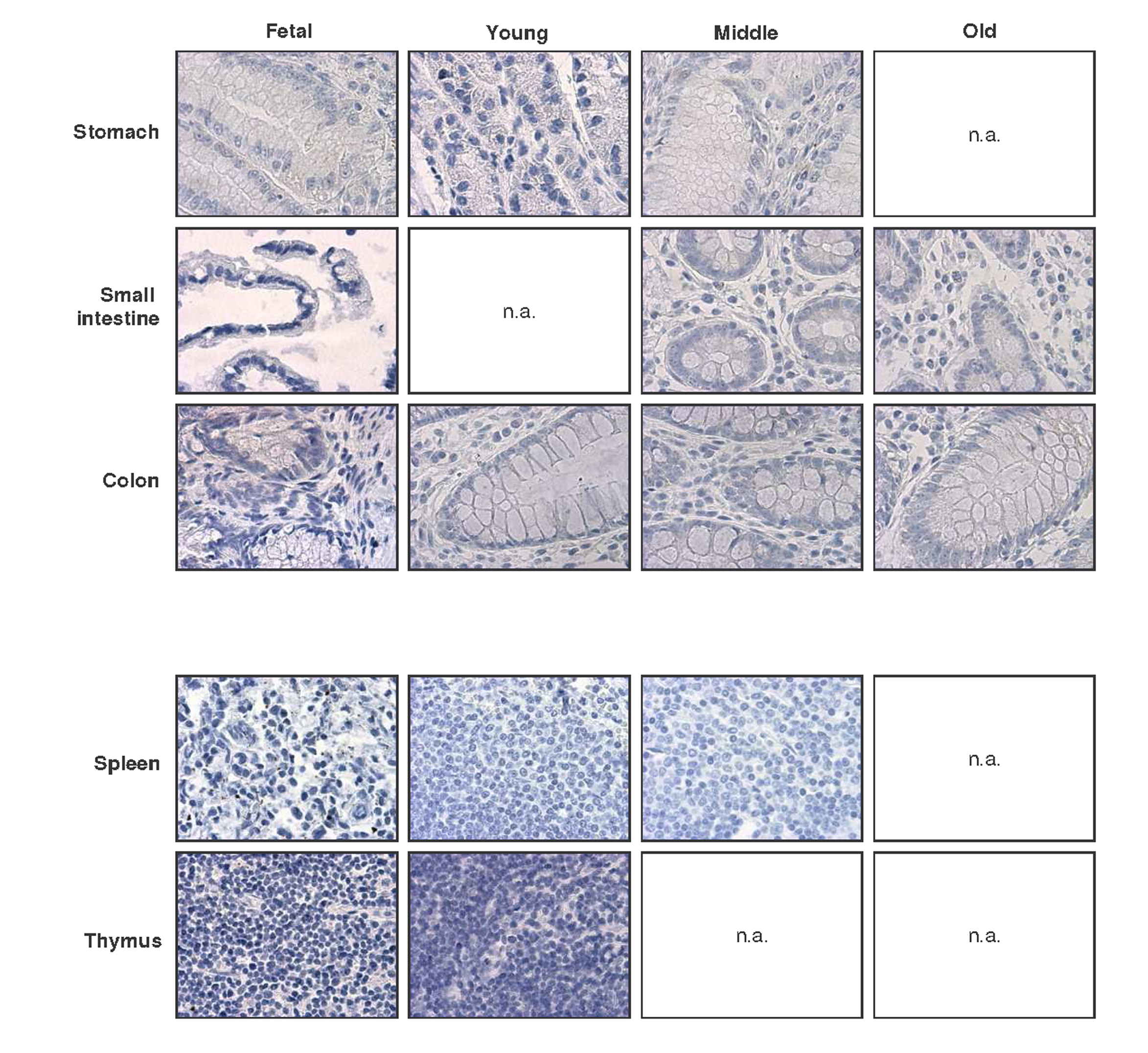

Supplement: Supplementary Figure 1 — All other steps were the same as those used to visualize HuR, AUF1, TIA-1, TTP (Figures 2-5) and prepare Tables 1-4. [file aging-01-681-s001.tif]

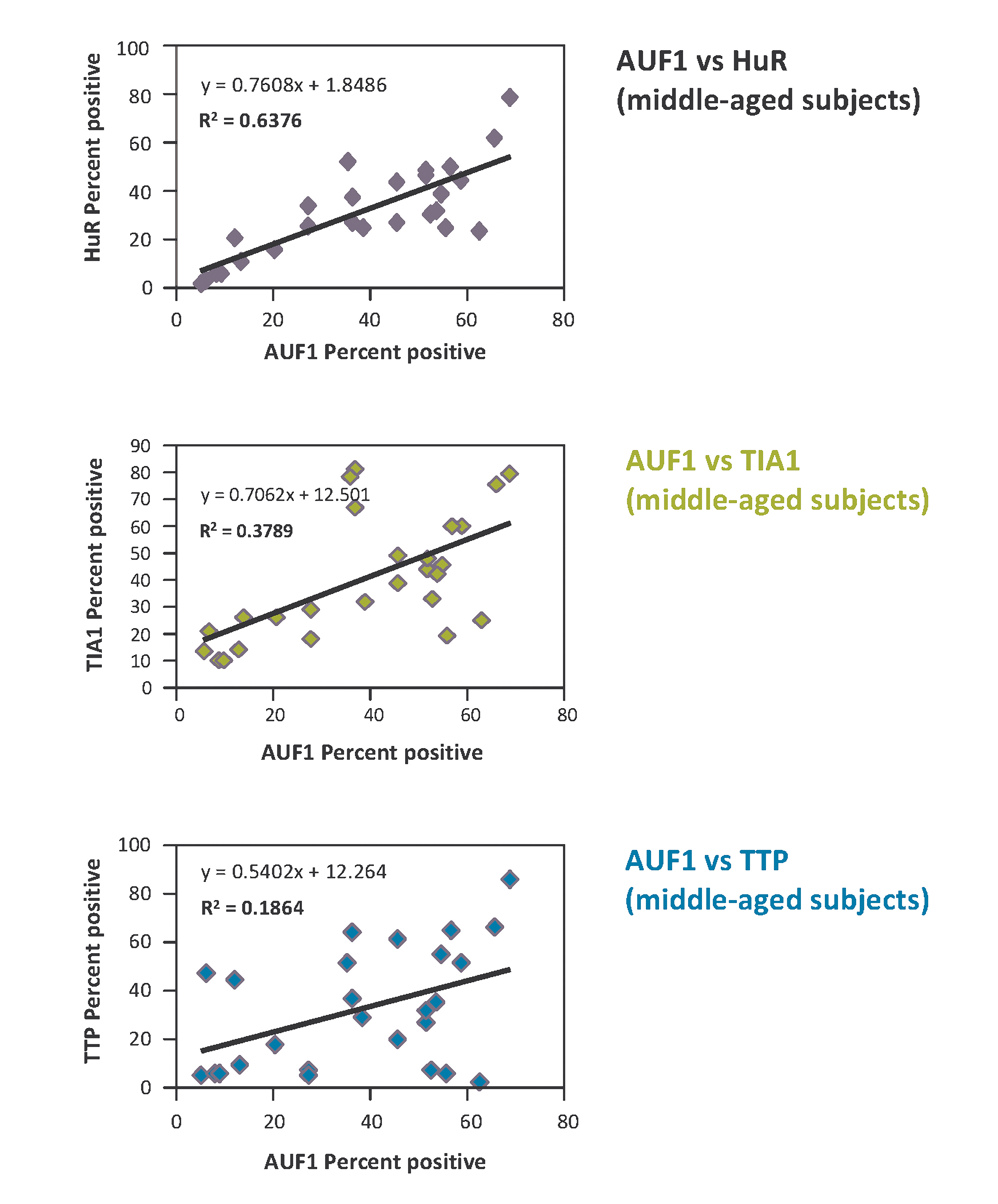

Supplement: Supplementary Figure 2 — Taking the middle-aged samples, the correlations between positive signals within a tissue were compared. Correlation coeficients (R2) indicate that the strongest correlation was seen between HuR and AUF1. In other age groups, AUF1 and HuR also correlated most strongly (not shown). [file aging-01-681-s002.tif]
