# Supplementary material for: Tissue- and age-dependent expression of RNA-binding proteins that influence mRNA turnover and translation
Source: Aging (Albany NY). 2009 Jul 26;1(8):681–98. doi: 10.18632/aging.100073 (PMC2806049; doi:10.18632/aging.100073)
Supplement: Supplementary Table 1 — (M), male; (F), female. y, years old. [file aging-01-681-s001.doc]

| Tissue | Fetal |  | Adult | | |  | |
| --- | --- | --- | --- | --- | --- | --- | --- |
| Adrenal Gland |  | 16y (M) | 37y (F) | 61y (F) |  | |  |
| Bladder | 28w (F) | 21y (F) | 28y (M) | 45y (M) | 51y (F) | |  |
| Bone Marrow |  | 16y (M) |  |  |  | |  |
| Eye |  | 1h (F) | 72y (F) |  |  | |  |
| Breast |  | 37y (F) | 37y (F) | 48y (F) |  | |  |
| Cerebellum | 37w (F) | 3m (M) | 3y (M) | 26y (M) | 45y (M) | |  |
| Cerebral Cortex | 37w (F) | 3m (M) | 3y (M) | 16y (M) | 26y (M) | |  |
| Fallopian Tube |  | 22y (F) | 22y (F) | 30y (F) |  | |  |
| Esophagus |  | 49y (M) | 51y (M) | 65y (M) |  | |  |
| Stomach | 22w (M) | 6y (M) | 24y (M) | 47y (M) | 51y (M) | |  |
| Small Intestine | 22w (F) | 37y (M) | 55y (M) | 64y (M) | 70y (M) | |  |
| Colon | 37w (F) | 8y (M) | 24y (M) | 36y (M) | 85y (F) | |  |
| Rectum |  | 56y (M) | 56y (M) | 70y (M) |  | |  |
| Heart | 16w (F) | 1h (F) | 16y (M) | 46y (M) | 59y (M) | |  |
| Kidney | 20w (F) | 30y (M) | 62y (F) | 67y (M) | 77y (M) | |  |
| Liver | 12w (M) | 30y (M) | 43y (M) | 56y (M) | 80y (F) | |  |
| Lung | 22w (F) | 24y (M) | 60y (M) | 67y (M) | 69y (M) | |  |
| Ovary |  | 23y (F) | 41y (F) | 50y (F) |  | |  |
| Pancreas |  | 16y (M) | 46y (M) | 49y (F) |  | |  |
| Parathyroid |  | 37y (M) |  |  |  | |  |
| Pituitary Gland |  | 54y (M) | 55y (M) |  |  | |  |
| Prostate |  | 72y (M) | 74y (M) | 75y (M) |  | |  |
| Skin | 28w (F) | 61y (F) | 62y (F) | 83y (F) |  | |  |
| Spinal Cord |  | 1h (F) | 46y (M) |  |  | |  |
| Spleen | 26w (M) | 12y (M) | 24y (M) | 49y (M) |  | |  |
| Striated Muscle | 28w (F) | 16y (M) | 24y (M) | 46y (M) | 62y (F) | |  |
| Testis |  | 16y (M) | 69y (M) | 73y (M) |  | |  |
| Thymus | 29w (M) | 1y (M) | 2.5y (F) | 13y (M) | 24y (M) | |  |
| Thyroid |  | 16y (M) | 37y (M) | 60y (F) |  | |  |
| Tongue | 20w (M) | 26y (M) |  |  |  | |  |
| Tonsil |  | 9y (F) | 10y (M) | 13y (M) |  | |  |
| Ureter |  | 2y (F) | 30y (M) | 46y (M) |  | |  |
| Uterus-Cervix |  | 29y (F) | 41y (F) | 44y (F) |  | |  |
| Uterus-Endometrium |  | 38y (F) | 40y (F) | 47y (F) |  | |  |
